# Structure-Based Drug Design of an Inhibitor of the SARS-CoV-2 (COVID-19) Main Protease Using Free Software: A Tutorial for Students and Scientists

**DOI:** 10.26434/chemrxiv.12791954

**Published:** 2020-08-12

**Authors:** Sheng Zhang, Maj Krumberger, Michael A. Morris, Chelsea Marie T. Parrocha, James H. Griffin, Adam G. Kreutzer, James S. Nowick

**Affiliations:** a Department of Chemistry, University of California, Irvine, Irvine, California 92697-2025, United States; b Department of Pharmaceutical Sciences, University of California, Irvine, Irvine, California 92697-2025, United States

## Abstract

This paper describes the structure-based design of a preliminary drug candidate against COVID-19 using free software and publicly available X-ray crystallographic structures. The goal of this tutorial is to disseminate skills in structure-based drug design and to allow others to unleash their own creativity to design new drugs to fight the current pandemic. The tutorial begins with the X-ray crystallographic structure of the main protease (M^pro^) of the SARS coronavirus (SARS-CoV) bound to a peptide substrate and then uses the UCSF Chimera software to modify the substrate to create a cyclic peptide inhibitor within the M^pro^ active site. Finally, the tutorial uses the molecular docking software AutoDock Vina to show the interaction of the cyclic peptide inhibitor with both SARS-CoV M^pro^ and the highly homologous SARS-CoV-2 M^pro^. The supporting information (supplementary material) provides an illustrated step-by-step guide for the inhibitor design, to help readers design their own drug candidates for COVID-19 and the coronaviruses that will cause future pandemics. An accompanying preprint in bioRxiv [https://doi.org/10.1101/2020.08.03.234872] describes the synthesis of the cyclic peptide and the experimental validation as an inhibitor of SARS-CoV-2 M^pro^.

## Introduction

SARS-CoV-2 is a highly infectious virus that causes COVID-19, a serious respiratory infection that has caused over 20 million infections and over 730,000 deaths worldwide, as of 08/11/20.^1^ SARS-CoV-2 causes infected cells to express a main protease (M^pro^ or 3CL protease) that is responsible for site-specifically cleaving the polyprotein, which is translated from viral mRNA within human cells. The proteolytic activity of M^pro^ is essential for the virus to generate the individual proteins that are necessary for replication and infection. The essential role of M^pro^, as well as the success of HIV protease inhibitors in the treatment of HIV/AIDS, make M^pro^ an attractive therapeutic target to treat COVID-19.^2–7^

**Figure 1.**
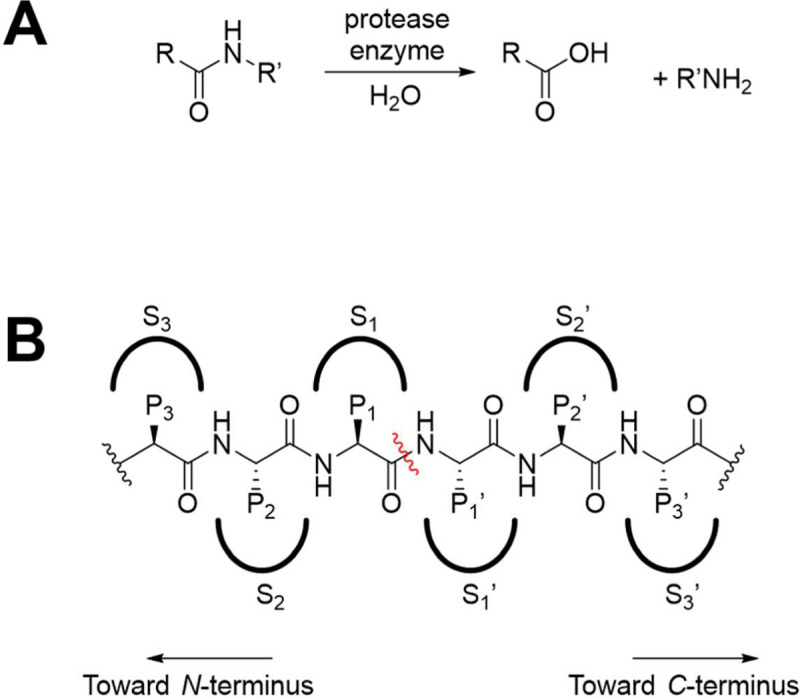
A: Amide bond hydrolysis by a protease enzyme. B: Binding of a protease to a polypeptide substrate. The side chains of the protein (P1, P2, P3, etc. and P1’, P2’, P3’, etc.) fit into pockets of the enzyme (S1, S2, S3, etc. and S1’, S2’, S3’, etc.). The scissile bond is designated with a wavy red line.

Proteases are enzymes that cleave polypeptide chains, hydrolyzing an amide bond within the polypeptide chain. Once the polypeptide is bound within the active site of the protease, the scissile amide bond is hydrolyzed to generate a carboxylic acid and amine (Figure 1A). The binding pockets of a protease are referred to as subsites, denoted by “S”. Typically, each subsite interacts with a specific side chain of the polypeptide substrate, denoted by “P”. The position at which the polypeptide substrate is cleaved determines the assignment of prime or no-prime notation. Prime notation refers to the *C*-terminal side and no-prime notation refers to the *N*-terminal side of the polypeptide and corresponding pockets (Figure 1B).

**Figure 2.**
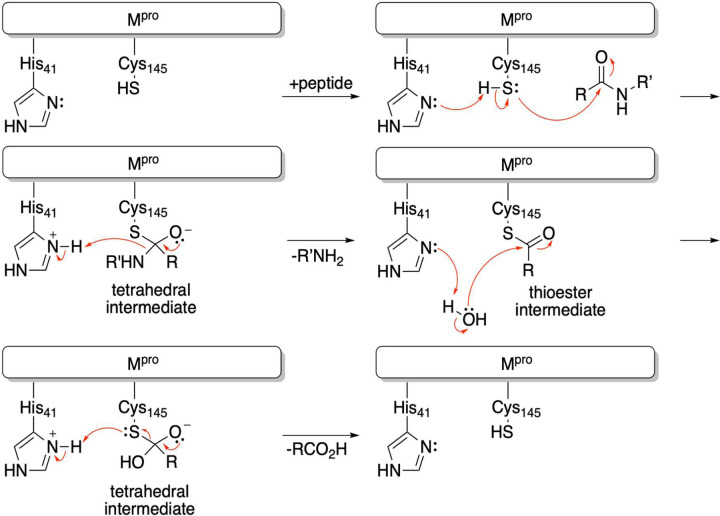
Proteolysis mechanism by the catalytic dyad of M^pro^.

SARS-CoV-2 M^pro^ is a member of the class of enzymes called cysteine proteases. These proteases usually contain a catalytic dyad of cysteine and histidine residues in the active site, which catalyze the cleavage of polypeptides, as shown below. The histidine deprotonates the cysteine thiol to give a nucleophilic thiolate, which adds to the amide carbonyl of the substrate to form a tetrahedral intermediate. The tetrahedral intermediate then breaks down to give a thiol ester and an amine. The electrophilic thiol ester is then hydrolyzed by water to give a carboxylic acid, thus completing the cleavage of the polypeptide substrate and regenerating the active enzyme (Figure 2).

In this tutorial, we will use the X-ray crystallographic structure of the homologous SARS-CoV M pro bound to a protein substrate to recapitulate the design of a cyclic peptide inhibitor of the SARS-CoV-2 M^pro^.^8^ We will first use the molecular modeling software UCSF Chimera to visualize the X-ray crystallographic structure of the SARS-CoV M^pro^ bound to the protein substrate.^9^ We will then modify the protein substrate to create a model of the cyclic peptide inhibitor within the SARS-CoV M^pro^. Finally, we will use AutoDock Vina to evaluate this model, by docking the inhibitor to SARS-CoV M^pro^ and then to SARS-CoV-2 M^pro^.^10^ We have selected these software packages, because they can be downloaded without cost and are easy to learn.^11–13^ These and other molecular modeling studies helped our laboratory decide to pursue the synthesis of the cyclic peptide and experimentally evaluate its promise as an inhibitor of SARS-CoV-2 M^pro^. In an accompanying preprint in bioRxiv, we describe the synthesis of the cyclic peptide and the experimental validation as an inhibitor of SARS-CoV-2 M^pro^.^8^

Here, we provide the rationale and then overview the process of designing the inhibitor with UCSF Chimera and evaluating it with AutoDock Vina. In the supporting information (SI), we provide an illustrated step-by-step protocol to teach others how to execute the design process. We anticipate this tutorial will help students and scientists use free software to design their own drug candidates for COVID-19 and the coronaviruses that will cause future pandemics.

## Results

### Selecting a starting structure for inhibitor design.

The design of the cyclic peptide inhibitor begins with the X-ray crystallographic structure of SARS-CoV M^pro^ (C145A) [Protein Data Bank (PDB) ID: 5B6O].^14^ The SARS-CoV M^pro^ is 96% identical to the SARS-CoV-2 M^pro^, and thus provides a good starting point for the design of inhibitors of SARS-CoV-2 M^pro^.^7^ In this crystal structure, the *C*-terminal fragment of one M^pro^ molecule extends into the active site of an adjacent M^pro^ molecule. The *C*-terminal fragment would normally be cleaved by SARS-CoV M^pro^, and thus the inactive C145A mutant provides a snapshot of the enzyme bound to one of its substrates. Molecules that mimic the *C*-terminal fragment, but are resistant to proteolysis, may serve as inhibitors that block viral replication.

### Modifying the *C*-terminal fragment of SARS-CoV M^pro^ to create a cyclic peptide inhibitor.

**Figure 3.**
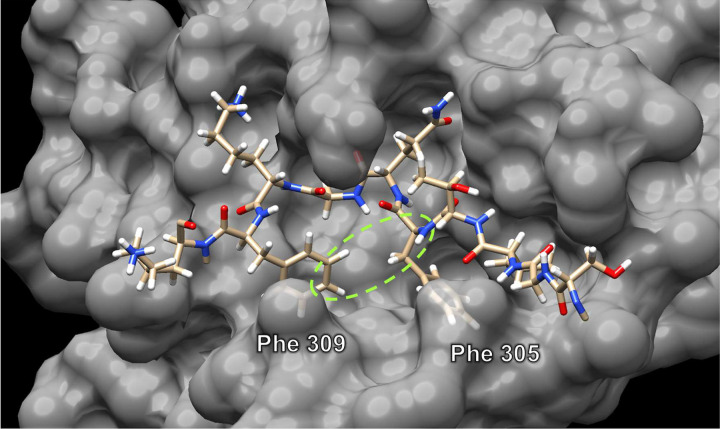
The interaction between the substrate (sticks) and the active site of the protein (grey surface). The green oval illustrates the concept of connecting the phenyl group of Phe 309 to the backbone of Phe 305.

We begin the tutorial by displaying the *C*-terminal fragment of the M^pro^ (substrate) as sticks and the adjacent M^pro^ protein as a van der Waals surface, to visualize how the substrate fits into the binding pockets of the protein active site. The substrate adopts a kinked conformation, in which the phenyl group of Phe 309 points toward the backbone of Phe 305. The proximity of Phe 309 and Phe 305 inspired us to connect the phenyl group of the Phe 309 with the backbone of Phe 305 to form a cyclic peptide (Figure 3). By cyclizing the linear substrate, we aim to lock the peptide substrate into its bound conformation and increase its stability toward proteolysis. Furthermore, cyclic peptides often exhibit greater cell permeability than the corresponding linear analogues, which is critical because M^pro^ constitutes an intracellular target.^15–20^

**Figure 4.**
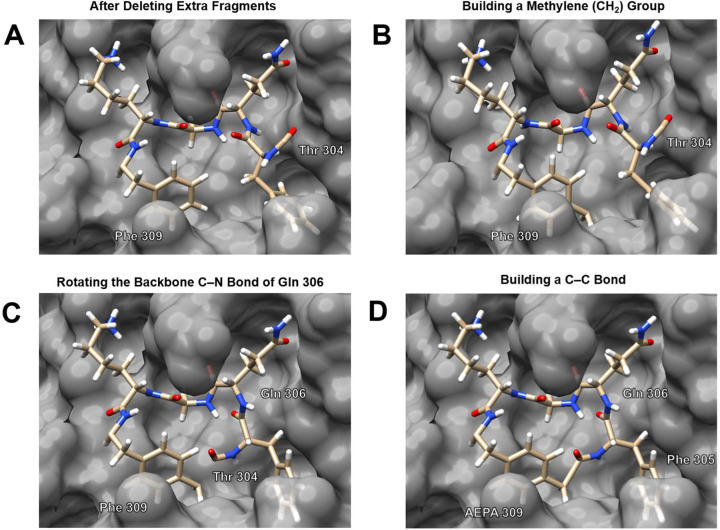
Building the cyclic peptide. A: The structure of the substrate after deleting extraneous fragments. B: Adding a CH_2_ group at the *para* position of Phe 309. C: Rotating the backbone Cα–N bond of Gln 306 to bring the Thr 304 carbonyl carbon close to the CH_2_ group. D: Building a C–C bond between the Thr 304 carbonyl carbon and the CH_2_ carbon.

To create the cyclic peptide, we delete Ser 301, Gly 302, Val 303, Thr 304 (except for the carbonyl group), Lys 310, and the carbonyl group of Phe 309, as these fragments are not needed in the cyclic peptide (Figure 4A). We then add a methylene (CH_2_) group at the *para* position of Phe 309 by building a tetrahedral methyl group (CH_3_) in UCSF Chimera and then deleting one of the hydrogen atoms of the methyl group (Figure 4B).

We next prepare to connect the Thr 304 carbonyl carbon to the newly built CH_2_ group, and thus cyclize the substrate. In UCSF Chimera, when the new bond is formed, it must not cross other atoms or bonds, otherwise subsequent structural minimization will fail. We rotate the backbone Cα–N bond of Gln 306 to bring the Thr 304 carbonyl carbon close to the CH_2_ group, to avoid crossing other atoms or bonds when building the new C–C bond (Figure 4C). We cyclize the substrate by building a C–C bond between the Thr 304 carbonyl carbon and the CH_2_ carbon. In cyclizing the substrate, we have built an unnatural amino acid residue — [4-(2-aminoethyl)phenyl]-acetic acid (AEPA) — from Phe 309 and Thr 304. The resulting cyclic peptide contains a β-turn comprising Phe 305 and Gln 306 (Figure 4D). We envision that hydrogen bonding within this β-turn might provide additional conformational rigidity to the cyclic peptide.

### Geometry optimization of the cyclic peptide inhibitor.

**Figure 5.**
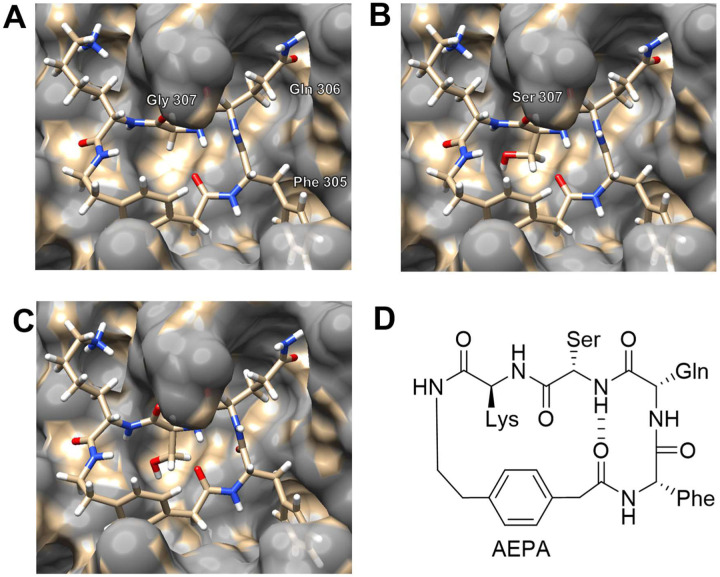
Geometry optimization of the cyclic peptide inhibitor. A: The structure of the Gly 307 cyclic peptide after geometry optimization.^21^ B: Gly 307 has been mutated to Ser. C: The structure of the Ser 307 cyclic peptide inhibitor after geometry optimization. D: The chemical structure of the Ser 307 cyclic peptide inhibitor.

At this point, the bond lengths, angles, and dihedral angles of the newly built cyclic peptide are not optimal. We are now ready to allow the cyclic peptide to relax to a low-energy conformation (local minimum) within the active site of the SARS-CoV M^pro^. We use the “minimize structure” tool to optimize the geometry of the cyclic peptide while holding the structure of M^pro^ fixed.^21^ The minimized structure (Figure 5A) has more reasonable bond lengths, angles, and dihedral angles than the structure prior to minimization (Figure 4D), with Phe 305 and Gln 306 forming a hydrogen-bonded β-turn.

To introduce additional conformational rigidity, we mutate Gly 307 to Ser, which is the most common residue at the P1’ position of SARS-CoV-2 M^pro^ substrates (Figure 5B). UCSF Chimera allows this point mutation to be achieved with a single command. After the point mutation, we perform a second round of geometry optimization to clean up the structure and afford a hypothesized structure of the cyclic peptide inhibitor (Figure 5C). Figure 5D illustrates the chemical structure of the cyclic peptide inhibitor, which we term UCI-1 (University of California, Irvine Coronavirus Inhibitor-1).^8^

### Docking the inhibitor to SARS-CoV M^pro^ and SARS-CoV-2 M^pro^.

In structure-based drug design, we would typically now synthesize the cyclic peptide inhibitor and evaluate its activity experimentally through studying its ability to block the cleavage of a fluorogenic peptide substrate by SARS-CoV-2 M^pro^. We would also attempt to co-crystalize the inhibitor with the M^pro^ to experimentally evaluate the structure hypothesized in Figure 5C. Using the co-crystal structure and additional structure-activity studies, we would then carry out iterative rounds of modification and optimization of the cyclic peptide inhibitor to achieve higher affinity and specificity for SARS-CoV-2 M^pro^.

Since this is exclusively a computational tutorial, we will use the molecular docking software AutoDock Vina in place of these experimental studies. UCSF Chimera enables AutoDock Vina to be used as a plugin, which allows us to conveniently perform molecular docking and view the docking results in UCSF Chimera.^22^ We will first evaluate the ability of the cyclic peptide inhibitor to bind the SARS-CoV M pro in silico and thus test our cyclic peptide inhibitor design. We will then evaluate the ability of the cyclic peptide inhibitor to bind SARS-CoV-2 M^pro^ in silico to test our inhibitor against the relevant target of COVID-19.

**Figure 6.**
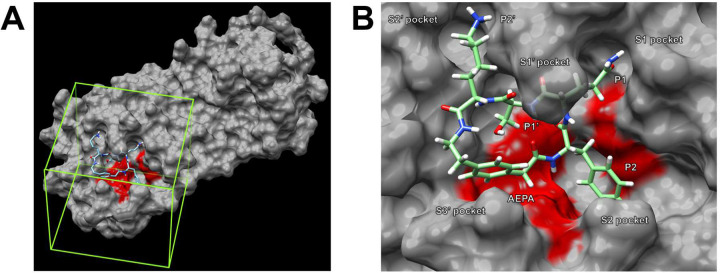
Molecular docking of the geometry-optimized cyclic peptide inhibitor to SARS-CoV M^pro^. A: The region to which AutoDock Vina will perform molecular docking is defined using a grid box encompassing the active site of SARS-CoV M^pro^. B: After molecular docking, the lowest energy conformation of the cyclic peptide inhibitor fits in the active site of SARS-CoV M^pro^.

In the first molecular docking exercise, we dock the geometry-optimized cyclic peptide inhibitor to the SARS-CoV M^pro^ structure (PDB 5B6O), which we have already used for the inhibitor design.^14^ We start by defining a receptor search region to which AutoDock Vina will dock the inhibitor. The receptor search region should thus include the active site of the SARS-CoV M^pro^. To facilitate identification of the active site, we highlight several residues in the active site in red (Cys 38, Cys 44, Met 49, Met 165, and His 41) and then set a grid box which engulfs all of the active site as the search region (Figure 6A). After the molecular docking is complete, we get five docked structures, with energy scores of −10.5, −8.0, −7.8, −7.7, and −7.6 kcal/mol. In the lowest energy structure, the inhibitor fits well in the active site of SARS-CoV M^pro^. The P2 (Phe), P1 (Gln), P1’ (Ser), and P2’ (Lys) side chains of the inhibitor occupy the S2, S1, S1’, and S2’ pockets, and the AEPA residue occupies the S3’ pocket (Figure 6B). This docking result demonstrates that the cyclic peptide inhibitor has the potential to bind to SARS-CoV M^pro^.

**Figure 7.**
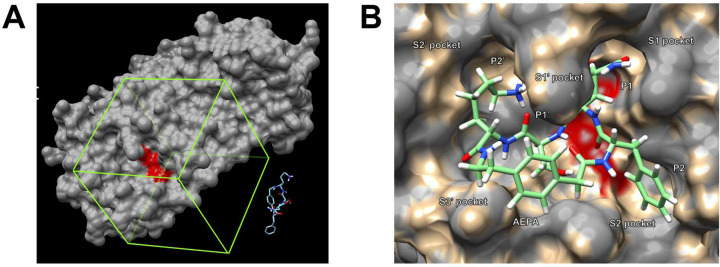
Molecular docking of the geometry-optimized cyclic peptide inhibitor to SARS-CoV-2 M^pro^. A: The region to which AutoDock Vina will perform molecular docking is defined using a grid box encompassing the active site of SARS-CoV-2 M^pro^. B: After molecular docking, the second lowest energy conformation of the cyclic peptide inhibitor fits in the active site of SARS-CoV-2 M^pro^.

In the second molecular docking exercise, we dock the geometry-optimized cyclic peptide inhibitor to a recently published crystal structure of SARS-CoV-2 M^pro^ (PDB 6YB7).^23^ We load the SARS-CoV-2 M^pro^ structure using the “fetch PDB” function in UCSF Chimera, and conduct molecular docking in a similar fashion to the previous exercise (Figure 7A). After the molecular docking is complete, we get ten docked conformations with energy scores of −8.1, −7.8, −6.8, −6.5, −6.5, −6.4, −6.4, −6.4, −6.2, and −5.6 kcal/mol. Although the lowest energy structure only partially fits into the active site of SARS-CoV-2 M^pro^, the second lowest energy structure of the inhibitor fits better in the active site. The P2 (Phe), P1 (Gln), P1’ (Ser), and P2’ (Lys) side chains of the inhibitor occupy the S1, S1’, S2, and S2’ pockets, while the AEPA residue sits near the S3’ pocket (Figure 7B). This docking result suggests that the cyclic peptide inhibitor that we designed based on SARS-CoV M^pro^ bound to a protein substrate might be repurposed to target SARS-CoV-2 M^pro^.

## Conclusions

UCSF Chimera and AutoDock Vina allow the structure-based design of inhibitors of SARS-CoV-2 M^pro^ as potential drug candidates. Using publicly available X-ray crystallographic structures and free software, anybody can unleash their imagination and try to invent new molecules that might help treat or prevent COVID-19 or other diseases. This tutorial demonstrates the process and provides a simple example of how a published X-ray crystallographic structure can be modified and manipulated with the goal of creating molecules to bind and block a critical enzyme. This tutorial can also be adapted to design inhibitors of other enzymes (e.g., HIV protease) from an X-ray crystallographic or NMR-based structure of an enzyme complex.^24–30^ We hope that this tutorial will help students and scientists design their own inhibitors of SARS-CoV-2 M^pro^ or other drug targets to help discover drugs for the treatment of COVID-19 and other diseases.
